# Different surgical approaches for the treatment of adjacent segment diseases after anterior cervical fusion: A retrospective study of 49 patients

**DOI:** 10.1097/MD.0000000000007042

**Published:** 2017-06-08

**Authors:** Feng Wang, Peng Wang, De-Chao Miao, Wei Du, Yong Shen

**Affiliations:** Department of Spine Surgery, Hebei Provincial Key Laboratory of Orthopedic Biomechanics, The Third Hospital of Hebei Medical University, Shijiazhuang, China.

**Keywords:** adjacent segment diseases, anterior cervical discectomy and fusion, anterior cervical fusion, dysphagia, Zero-profile

## Abstract

Studies in the literature have not delineated the surgical approaches of symptomatic adjacent segment diseases (ASDs) in patients undergoing reoperation after an initial anterior cervical fusion (ACF). The purpose of this study was to determine the optimal surgical approaches of ASD and the incidence of the dysphagia after reoperation.

This was a retrospective study of 49 patients with ASD after an initial ACF surgery, which had undergone a reoperation at our medical center between January 2010 and December 2014. The surgical approaches were used by anterior cervical discectomy and fusion (ACDF), ACDF with the Zero-profile device, laminoplasty, and laminectomy with internal fixation. Patients were classified according to the different surgical approaches of anterior (n = 38) versus posterior (n = 11) groups and ACDF (n = 25) versus Zero-P (n = 13) groups. Clinical evaluations were performed preoperatively and repeated in 24 months after operation.

This retrospective study included 26 men and 23 women with a mean age at revision surgery of 54.3 years and ASD onset time of 7.3 years. The patients were followed up with an average of 4.1 years. The reoperation rate was 5.4% in this study. The Japanese Orthopaedic Association (JOA), Neck Disability Index (NDI), and visual analogue scale (VAS) scores demonstrated significant improvements compared with preoperative in both anterior and posterior groups (*P* < .05). However, there were no differences between the 2 groups (*P* > .05). The operation time of ACDF group was more than Zero-P group, with significant differences (*P* < .05). However, there were no differences in JOA, NDI, and VAS scores between the ACDF and Zero-P groups pre- and postoperative (*P* > .05). A total of 12 (24.5%) patients had dysphagia after reoperation. The incidence of dysphagia in Zero-P group (1/13) was less than ACDF group (11/25), with significant differences (*P* < .05). There were no cases of major neurological or vascular complications, and wound complications.

The clinical situation, initial operation, and secondary preoperative imaging findings were analyzed comprehensively, anterior or posterior approach were chosen, which can effectively relieve spinal cord compression and improve spinal cord function. In ACDF with the Zero-profile device surgery, there was no need to remove the previous internal fixation, shorten the operation time, and reduce the incidence of postoperative dysphagia.

## 1 Introduction

Anterior cervical fusion (ACF), including anterior cervical discectomy and fusion (ACDF) and anterior cervical corpectomy and fusion (ACCF), is widely accepted as a standard surgical treatment for cervical spondylosis with radiculopathy or myelopathy refractory to conservative management.^[1,2]^ ACF allows direct decompression of the neural elements and generally accompanied by interbody fusion and anterior plate stabilization. However, biomechanical and clinical studies suggested that adjacent-level kinematic might predispose to adjacent-level degeneration after ACF.^[3,4]^ Degenerative changes in adjacent-level and unsatisfactory clinical outcomes were documented.^[5,6]^ Adjacent segment diseases (ASDs), whether representing an enhanced degeneration due to adjacent fusions or merely the natural progression of degeneration, have been recognized as an important entity after ACF.^[7–9]^ A seminal report by Hilibrand et al^[10]^ on symptomatic ASD in patients who had previously undergone ACDF procedures found that 2.9% of patients per year developed either new radiculopathy or myelopathy complaints. Chung et al^[11]^ investigated 177 patients and reported that radiographic and clinical adjacent-segment pathologies were found in 92.1% and 19.2%; approximately 7% patient needed follow-up surgery. Recently, Lee et al^[12]^ reported adjacent segments underwent surgical treatment at an annual rate of 2% after cervical fusion and predicted that 22% of patients would need a reoperation for symptomatic ASD within 10 years. To date, studies in the literature have not delineated the surgical approaches of symptomatic ASD in patients undergoing reoperation after an initial ACF.^[13–15]^

Dysphagia is increasingly recognized as a common complication after ACDF.^[16]^ A review of literature yields a largely varied incidence of postoperative dysphagia, ranged from 2% to 67%.^[17–20]^ However, the pathophysiology of dysphagia has not been well understood. A series of risk factors such as reoperation, long operation time, are associated with an increase in postoperative dysphagia incidence. It has been reported that the Zero-profile device lead to similar clinical and radiographic outcomes compared with ACDF with plating and carry a lower risk of postoperative dysphagia.^[21,22]^ However, there are few studies about ACDF with the Zero-profile device, which was taken as a revision surgery for ASD after primary surgery, with the aim to reduce the incidence of dysphagia.

The patients of this study underwent a reoperation for ASD after ACF were retrospectively analyzed. According to the clinical situation, initial operation and secondary preoperative imaging findings were analyzed comprehensively, the surgical approaches were used by ACDF, ACDF with the Zero-profile device, laminoplasty, and laminectomy with internal fixation. The goal of this study was to determine the surgical approaches of symptomatic ASD in patients undergoing reoperation after an initial ACF, and the incidence of the dysphagia after the revision surgery.

## 2 Materials and methods

### 2.1 Study population selection

**Figure 1:**
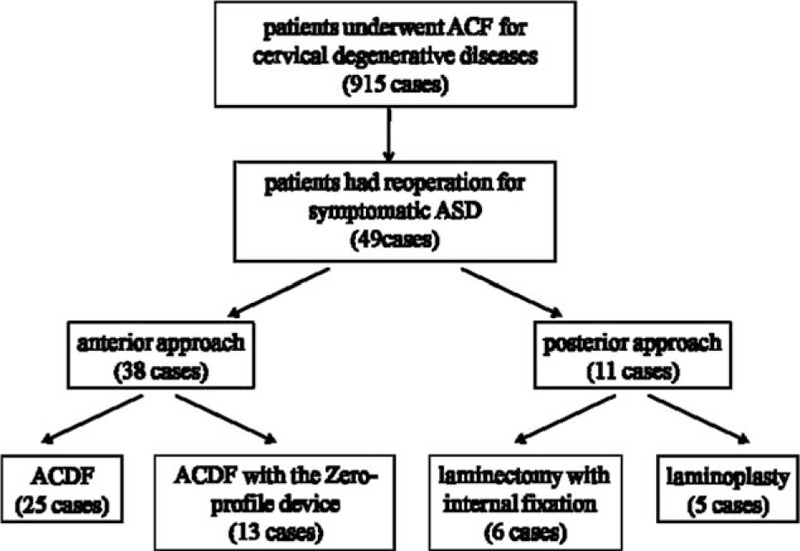
Schematic of patients had a revision surgery for symptomatic ASD after an initial ACF. ASD = adjacent segment disease, ACF = anterior cervical fusion.

From January 2000 to December 2009, a total of 915 patients underwent ACF for cervical radiculopathy and myelopathy in the authors’ institution. Forty-nine patients were identified who had a revision surgery for symptomatic ASD between January 2010 and December 2014 (Fig. 1). All the patients had no expression of already existing degeneration (radicular or myelopathic signs and symptoms that correlate with imaging evidence of degeneration) at the time of the first surgery. Furthermore, these patients who developed gradual neurological changes followed 6 months of invalid conservative treatment. However, the patients with cervical spine trauma, tumor spinal pathologies, neoplasm, spinal infections, congenital deformations, and chronic systemic illnesses such as rheumatoid arthritis and neurodegenerative diseases were excluded in this study. This study had been approved by ethics committee of The Third Hospital of Hebei Medical University. There is no need to obtain informed consent from patients because this is a retrospective study and all data were collected and analyzed anonymously.

### 2.2 Surgical management

Before the reoperation, all of the patients were taken with x-ray (anterior-posterior, lateral, and flexion-extension), computed tomography (CT) (including sagittal reconstruction), and magnetic resonance imaging (MRI) scans. According to the clinical situation, initial operation and secondary preoperative imaging findings, the surgical approaches were used by ACDF, ACDF with the Zero-profile device, laminoplasty, and laminectomy with internal fixation. All patients received reoperation by the same senior surgeon. All patients underwent general anesthesia.

**Figure 2:**
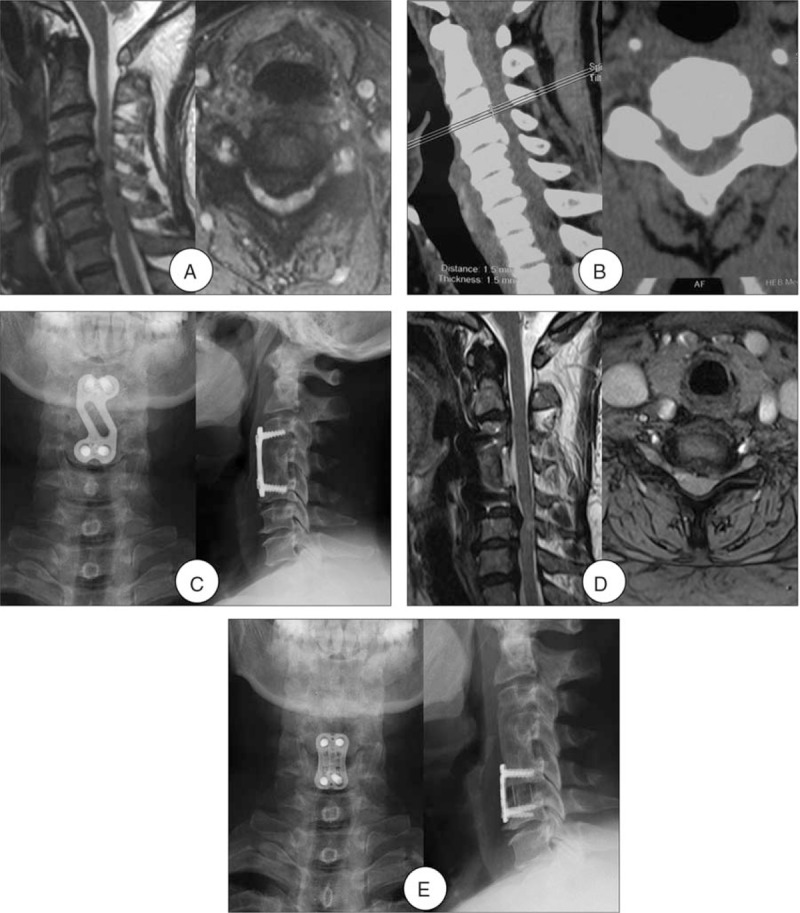
A 52-year-old woman developed one-level ASD 7 years after initial surgery. (A) Preoperative MRI of this patient shown severe compressions of the spinal cord at C4-5 level as well as high signal intensity in the spinal cord. (B) Preoperative CT shown ossification formation at C4-5. (C) Radiograph after initial surgery shown ACCF at C4. (D) MRI at 7-year follow-up shown development of ASD and spinal cord compression at C5–6. (E) Radiograph after the reoperation shown ACDF at C5-6 and the fixed plate of the initial surgery was removed. ACCF = anterior cervical corpectomy and fusion, ACDF = anterior cervical decompression and fusion, ASD = adjacent segment disease, CT = computed tomography, MRI = magnetic resonance imaging.

**Figure 3:**
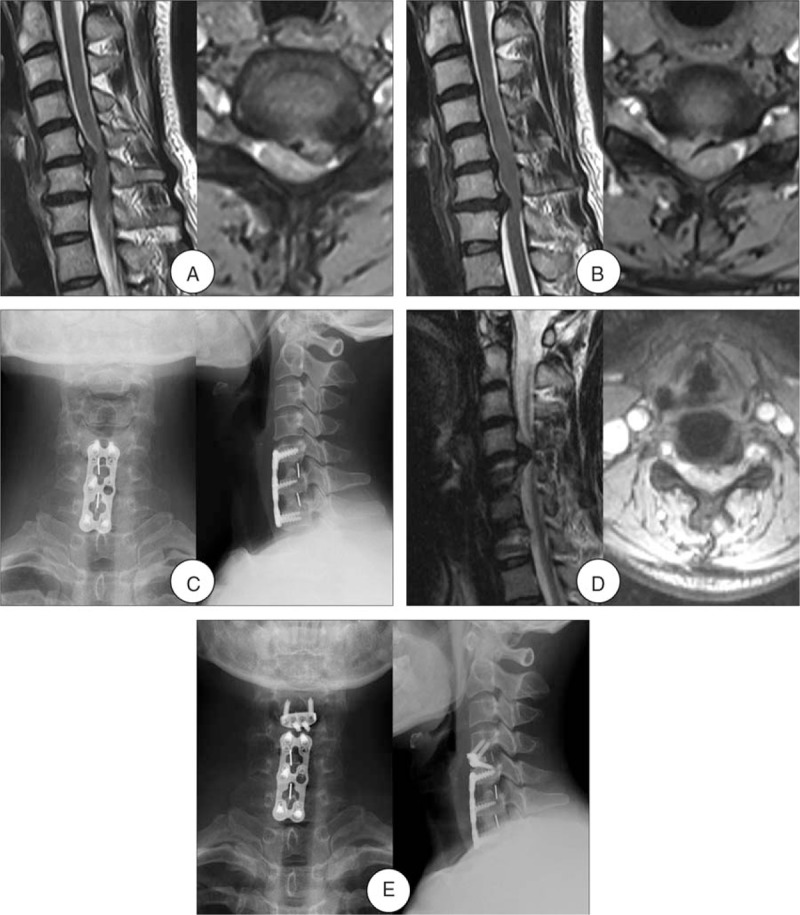
A 40-year-old woman developed one-level ASD 3 years after initial surgery. (A) and (B) Preoperative MRI of this patient shown severe compressions of the spinal cord at C5-6 and C6-7 levels. (C) Radiograph after initial surgery shown ACDF at C5-6 and C6-7. (D) MRI at 3-year follow-up shown development of ASD and spinal cord compression at C4–5. (E) Radiograph after the reoperation shown ACDF with the Zero-profile device at C4-5 and the fixed plate of the initial surgery was not removed. ACDF = anterior cervical decompression and fusion, ASD = adjacent segment disease, MRI = magnetic resonance imaging.

Patients who met the following criteria were included in the anterior approach group: the numbers of the reoperation segments were ≤2; no severe ossification of the posterior longitudinal ligament (OPLL) or ossification of the yellow ligament. The anterior approach group also included ACDF group and ACDF with the Zero-profile device (Zero-P group). The ACDF group should remove the fixed plate of the initial surgery (Fig. 2), whereas the Zero-P group not (Fig. 3).

**Figure 4:**
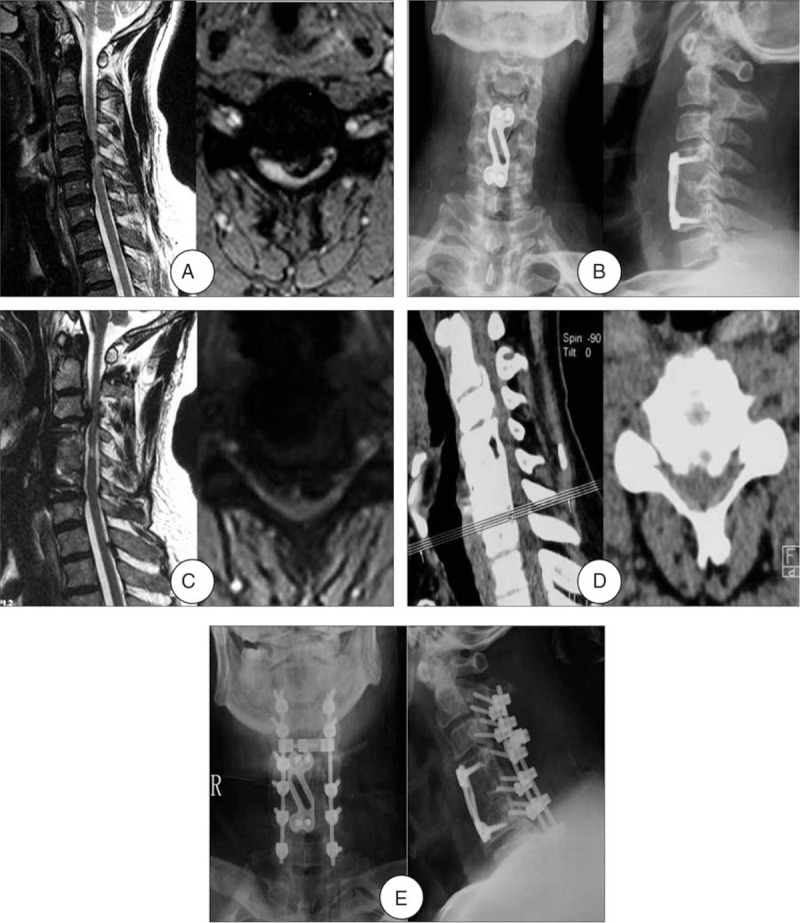
A 66-year-old man developed multi-level ASD 11 years after initial surgery. (A) Preoperative MRI of this patient shown severe compressions of the spinal cord at C4-5 level as well as high signal intensity in the spinal cord. (B) Radiograph after initial surgery shown ACCF at C4. (C) and (D) MRI and CT at 11-year follow-up shown development of ASD and spinal cord compression at C2-3, C3-4, and C6-7. (E) Radiograph after the reoperation shown laminectomy with internal fixation. ACCF = anterior cervical corpectomy and fusion; ASD = adjacent segment disease, CT = computed tomography, MRI = magnetic resonance imaging.

Patients who met the following criteria were enrolled in the posterior approach group receiving laminoplasty and laminectomy with internal fixation: the numbers of the reoperation segments were ≥3; the OPLL cannot be removed completely by the anterior approach. Among those patients, who with obvious cervical kyphosis, cervical instability, or severe ossification of the yellow ligament received laminectomy with internal fixation (Fig. 4), whereas others were treated with laminoplasty.

Ambulation was allowed on the second day after surgery, whereas external immobilization of the cervical spine was kept for 2 to 3 months with a cervical collar.

### 2.3 Evaluation criteria

Clinical data including clinical and radiological evaluation results were collected preoperatively and at 3, 6, 12, and 24 months after surgery. All patients were followed up for at least 2 year after surgery. The modified Japanese Orthopaedic Association (JOA) scoring system was used to determine functional status before surgery and at the final follow-up visit. The recovery rate (%) at the final follow-up visit was calculated by using the Hirabayashi method: (postoperative JOA score − preoperative score)/(17 − preoperative score) × 100%. Neck Disability Index (NDI) was used to evaluate how much the neck pain affected the ability to manage daily life. Visual analogue scale (VAS) was used to determine neck and arm pain before surgery and at the final follow-up visit. Patients enrolled in this study were evaluated for pre- and postoperative dysphagia by interview for subjective complaints, objective swallowing evaluation. We used Bazaz dysphagia score in which dysphagia was divided into 4 grades, none, mild, moderate, and severe (Table 1).^[17]^ Serious adverse events were those that could influence the clinical result, such as loosening of the implant, collapse of the fusion intervertebral space, hematoma, and deep infection. Successful fusion was defined as R4 degrees of angular motion on flexion and extension radiographs, the presence of bridging trabecular bone between the fused vertebrae, and the absence of any radiolucent zones spanning <50% of the implant-vertebral interface on CT images. Two independent radiologists assessed the radiographs. In the event of disagreement about fusion healing, a third independent reading was obtained.

**Table 1:**
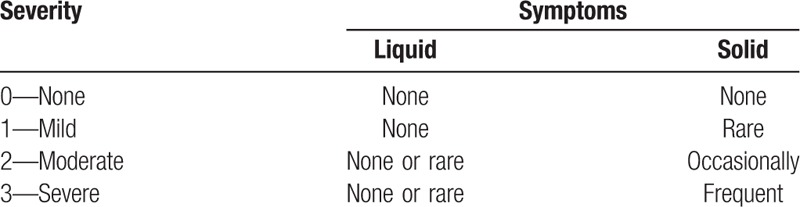
The Bazaz grading system for dysphagia.

### 2.4 Statistical methods

All data were collected, and the software of by SPSS Version 17.0 (SPSS Inc, Chicago, IL) was used for the statistical evaluation. Results were presented as mean ± SD. A paired *t* test was used to identify a significant difference between pre- and postoperative measurements of JOA, NDI, and VAS for neck and arm pain for each group. The independent 2-sample *t* test was used to identify a significant difference between the groups. Categorical data (dysphagia ratio) were compared via the chi-square test (Fisher exact test for small samples). In all analyses, *P* value <.05 was considered statistically significant.

## 3 Results

This retrospective study included 49 patients consisting of 26 men and 23 women with a mean age at revision surgery of 54.3 ± 8.2 (41–76) years and ASD onset time of 7.3 ± 3.7 (2–15) years. After the reoperation, the patients were followed up with an average of 4.1 years (rang 2–6 years). The reoperation rate was 5.4% in this study. Among them, 34 patients had a single primary fused level, 12 patients had 2 levels, and 3 patients had ≥3 levels. ASD occurred superior to the prior fusion in 24 patients, inferior in 19 patients, and at both adjacent levels in 6 patients. The adjacent levels were located at C3-4 in 9 patients, C4-5 in 22 patients, C5-6 in 20 patients, C6-7 in 23 patients, C7-T1 in 3 patients.

Of these 49 patients, 38 patients were in the anterior group and 11 patients were in the posterior group. There were 25 patients in the ACF group and 13 patients in the Zero-P group. No significant differences existed in age, sex, primary fused level, the onset time of ASD, adjacent level involved or follow-up between anterior and posterior groups (*P* < .05, Table 2). Moreover, no significant differences existed in age, sex, primary fused level, the onset time of ASD, adjacent level involved or follow-up between ACF and Zero-P groups (*P* < .05, Table 3). The recovery rate was 69.0% and 67.5% in patients who underwent anterior and posterior procedures, respectively. The operation time of anterior group [(74.1 ± 26.7) min] was less than posterior group [(121.5 ± 33.9) min], with significant differences (*P* < .05, Table 2). The JOA, NDI, and VAS scores demonstrated significant improvements compared to the preoperative scores in both anterior and posterior groups (*P* < .05). However, there were no differences between the 2 groups (*P* > .05, Table 4). The recovery rate was 67.9% and 68.2% in patients who underwent ACDF and ACDF with the Zero-profile device, respectively. The operation time of ACDF group [(81.2 ± 28.3) min] was more than Zero-P group [(60.4 ± 25.6) min], with significant differences (*P* < .05, Table 3). However, there were no differences in preoperative JOA, NDI, and VAS scores between the ACDF and Zero-P groups (*P* > .05). Both groups reported significant improvements in JOA, NDI, and VAS scores from the preoperative means (*P* < .05). However, no differences were found between the groups (*P* > .05, Table 5). A total of 12 (24.5%) patients had dysphagia after the revision surgery. The incidence of dysphagia in anterior group (31.6%, 12/38) was more than posterior group (0, 0/11), with significant differences (*P* < .05, Table 4). Moreover, the incidence of dysphagia in Zero-P group (7.7%, 1/13) was less than ACDF group (44.0%, 11/25), with significant differences (*P* < .05, Table 5).

**Table 2:**
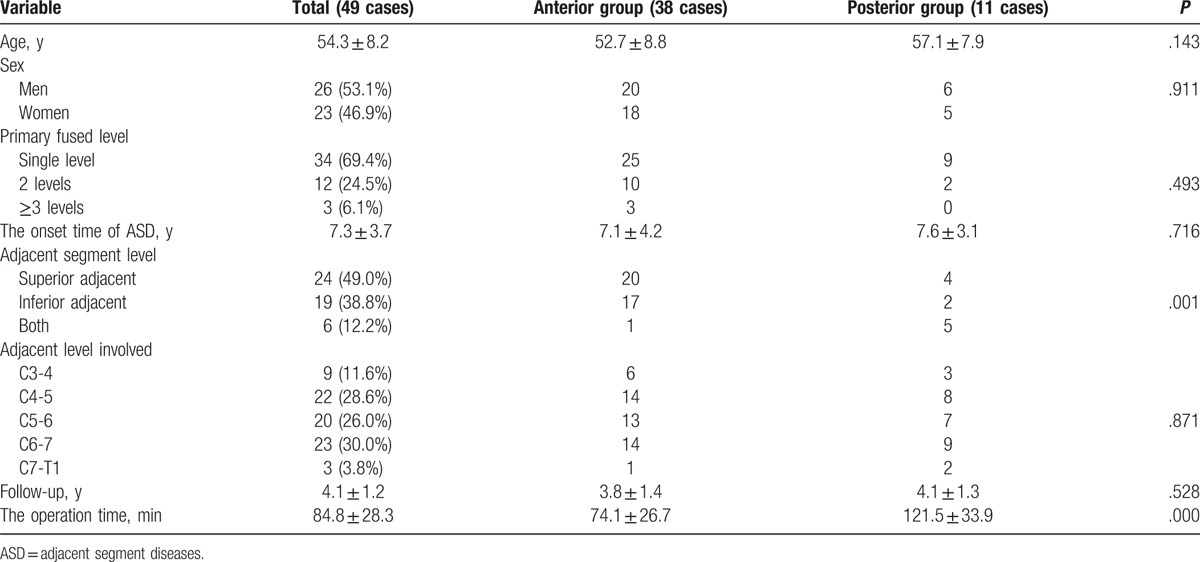
Patient demographics (anterior and posterior groups).

**Table 3:**
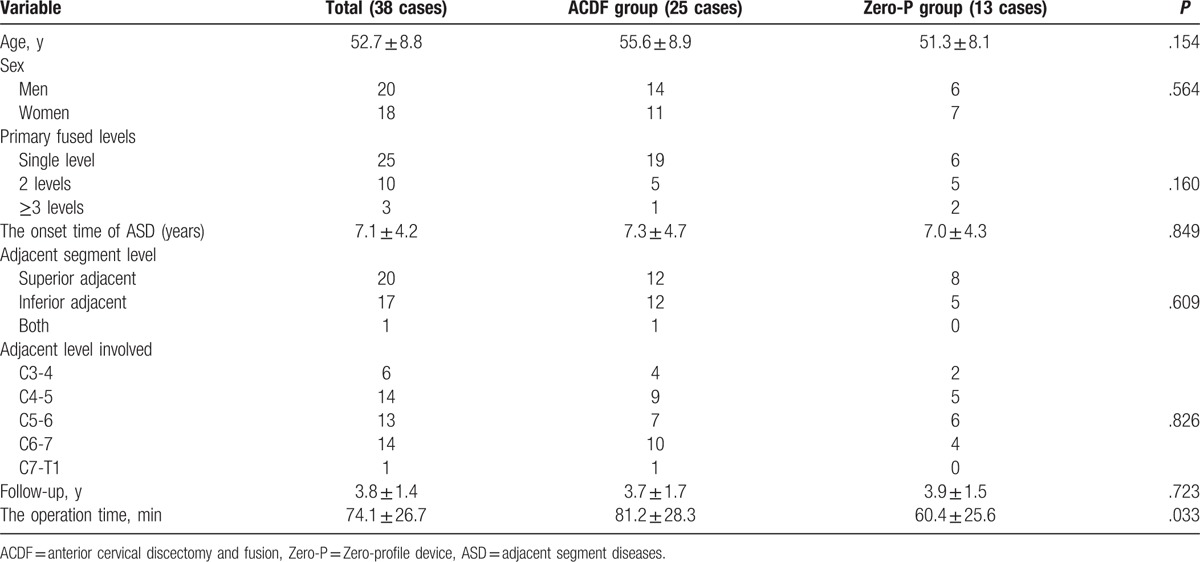
Patient demographics (ACDF and Zero-P groups).

**Table 4:**
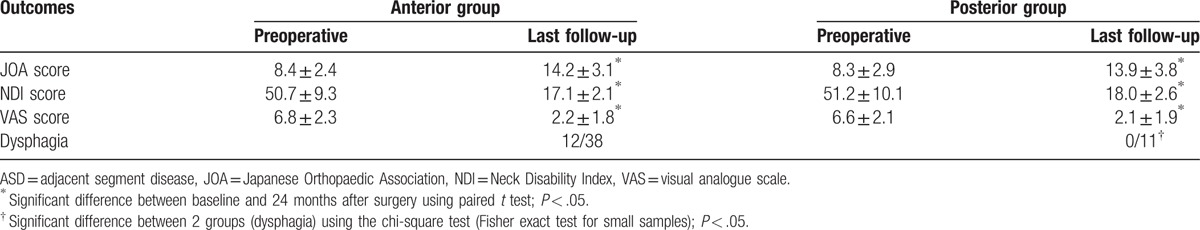
Comparison of surgical results between anterior and posterior group in patients with ASD.

**Table 5:**
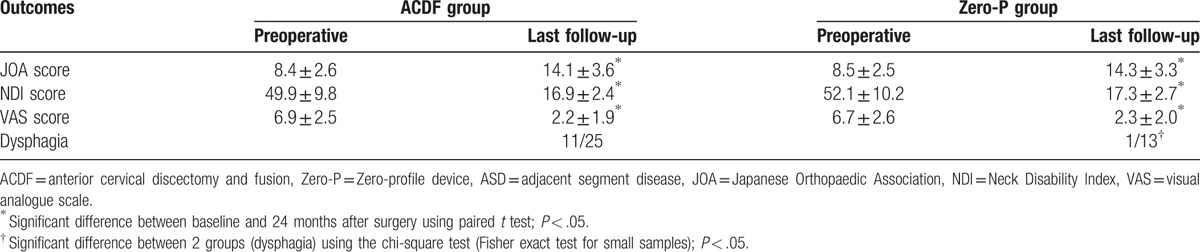
Comparison of surgical results between ACDF and Zero-P group in patients with ASD.

The radiographic examination showed imaging fusion among the patients in anterior group and laminectomy with internal fixation. Also, the patients underwent laminoplasty with open door condition maintained well. There were no cases of major neurological or vascular complications, and wound complications.

## 4 Discussion

ACF was first described in 1950s and had been widely performed for the treatment of cervical degenerative diseases. However, in the past few decades, an increasing number of studies and data showed that ASD after ACF had become a considerable challenge for surgeons. A review of the literature, the rate of reoperation of ASD patients undergoing ACF for cervical radiculopathy and myelopathy was ranged from 2.1% to 22%.^[9–12,23]^ These results are largely in line with the study by Hilibrand et al,^[9,10]^ which monitored ASD development after ACDF and found an incidence of 2.9% per year. During the 10 years, Hilibrand et al^[10]^ predicted that 25.6% of the patients will develop ASD, of which two-thirds required a revision surgery. In this long-term study, 49 (5.4%) patients were involved to investigate the outcomes of reoperation for ASD after ACF. The reoperation rate was similarly compared with previous long-term follow-up studies. In addition, this study found that the C4-5, C5-6, and C6-7 spinal levels were in the higher risk of developing ASD than others. The segmental distribution is consistent with the first surgery for cervical degenerative diseases.^[24]^ In addition to this, the ASD levels were more likely to occur at superior to the prior fusion. This is also consistent with previous findings.^[10,25]^ Komura et al^[26]^ reported that the ASD occurred less frequently among patients in whom C5-6 and C6-7 were fused than among those in whom C5-6 or C6-7 was left at an adjacent level. It suggested that the fusion might cause increased intradiscal pressure and ROM, which lead to accelerate adjacent segment degeneration.

Previous studies have demonstrated that the development of ASD may be influenced by several factors, including the age, smoking history, number and location of fusion segments, plate-to-disc distances, spinal canal stenosis, preexisting degenerative changes at adjacent segments, excessive disc space distraction and kyphotic malalignment.^[12,27–30]^ However, it is still unclear that whether it is the result of natural degeneration or caused by fusion. If ASD occurs, most of the patients should be treated conservatively. However, a revision surgery should be considered for the patients with obvious clinical manifestation and poor conservative treatment. At present, there is no gold standard treatment for ASD.^[30]^ The main reoperation methods reported in the literature are ACF, laminoplasty, laminectomy with internal fixation, and even artificial disc replacement.^[13–15,30–34]^ Furthermore, these procedures have achieved good short-term clinical results. However, there is currently no consensus on the choice of the reoperation procedures for ASD after ACF. In this study, according to the clinical situation, initial operation and secondary preoperative imaging findings were analyzed comprehensively, the surgical approaches were used by ACDF, ACDF with the Zero-profile device, laminoplasty and laminectomy with internal fixation. And this study achieved a good clinical efficacy. It suggested that the choice of reoperation procedures should be based on the following. Patients who met the following criteria were treated with anterior approach: the numbers of the reoperation segments were ≤2; no severe OPLL or ossification of the ligamentum flavum. But patients who met the following criteria were treated with laminoplasty and laminectomy with internal fixation: the numbers of the reoperation segments were ≥3; (2) the OPLL cannot be removed completely by the anterior approach. Among those patients, especially with obvious cervical kyphosis, cervical instability, or severe ossification of the ligamentum flavum received laminectomy with internal fixation, whereas others were treated with laminoplasty. In this study, according to the characteristics of the pressure, number of reoperation segments, cervical sagittal alignment and segmental mobility, and ossification of the ligamentum flavum, we chose different surgical approaches and obtained good results after reoperation. Also, there were no cases of major neurological or vascular complications, and wound complications during and after the reoperation.

Dysphagia is a common complication after ACDF, occurring with a frequency ranging from 2% to 67%, and most of the symptoms disappear within 3 months after the operation.^[16–20]^ At present, it is not clear about the mechanism of dysphagia after ACDF. The anterior cervical plate can increase interbody fusion rates and stability, restore or maintain cervical lordosis and prevent interbody graft subsidence or dislocation in ACDF surgery.^[35–38]^ However, anterior plating may also be associated with the dysphagia after ACDF. Previous studies have shown that the thickness of anterior cervical plate and the surrounding scar formation have a certain impact on the occurrence of dysphagia after ACDF.^[39,40]^ In ACDF surgery, it is necessary to pull the esophageal and trachea to one side. Many research confirmed that the tensile strength and time of esophageal are the important factors that influence the postoperative dysphagia.^[17,41]^ In this study, a total of 12 (24.5%) patients had dysphagia after the revision surgery. The incidence of dysphagia was high, and thus it was related to the revision surgery. The incidence of dysphagia in Zero-P group (7.7%) was less than ACDF group (44.0%). It suggested that ACDF often need to excessively expose the esophageal in the revision surgery because of separating the scar tissue, and providing enough space for plating and nailing. However, there was no need to overly pull the soft tissue in ACDF with the Zero-profile device surgery, so that the damage to the esophagus is less than ACDF group. Without using the anterior cervical plate, there was no the compression of plate and scar formation surrounding. In addition, the placement of Zero-profile device was simpler than that of anterior plate fixation, and the operation time was significantly shortened. This study through the comparison confirmed that the Zero-P group obtaining a same surgical effect, compared with ACDF group can significantly shorten the operation time, and reduce the incidence of dysphagia.

## 5 Conclusions

A revision surgery should be considered for the patients with obvious clinical manifestation and poor conservative treatment. The clinical situation, initial operation and secondary preoperative imaging findings were analyzed comprehensively, anterior or posterior approach were chosen, which can effectively relieve spinal cord compression and improve spinal cord function. In ACDF with the Zero-profile device surgery, there was no need to remove the previous internal fixation, shorten the operation time and reduce the incidence of postoperative dysphagia. However, this study was only a retrospective study with a small sample size to explore the revision surgery for ASD after ACF. Prospective multiple-center studies, long-term data, control group, and postoperative dysphagia formation are needed to confirm the result. Furthermore, cervical artificial disc replacement was not included in this study. In the future study, we can explore the risk factors for ASD after ACF.
